# How do prolonged anchorage-free lifetimes strengthen non-small-cell lung cancer cells to evade anoikis? – A link with altered cellular metabolomics

**DOI:** 10.1186/s40659-023-00456-z

**Published:** 2023-08-05

**Authors:** Rungroch Sungthong, Hnin Ei Ei Khine, Somruethai Sumkhemthong, Pithi Chanvorachote, Rossarin Tansawat, Chatchai Chaotham

**Affiliations:** 1 Department of Biochemistry and Microbiology, Faculty of Pharmaceutical Sciences, Chulalongkorn University, Bangkok, 10330 Thailand; 2 School of Pharmacy, Eastern Asia University, Pathum Thani, 12110 Thailand; 3 Department of Pharmacology and Physiology, Faculty of Pharmaceutical Sciences, Chulalongkorn University, Bangkok, 10330 Thailand; 4 Center of Excellence in Cancer Cell and Molecular Biology, Faculty of Pharmaceutical Sciences, Chulalongkorn University, Bangkok, 10330 Thailand; 5 Department of Food and Pharmaceutical Chemistry, Faculty of Pharmaceutical Sciences, Chulalongkorn University, Bangkok, 10330 Thailand

**Keywords:** Non-small-cell lung cancer, Anchorage-independent growth, Anoikis, Anoikis resistance, Metastasis, Metabolomics

## Abstract

**Background:**

Malignant cells adopt anoikis resistance to survive anchorage-free stresses and initiate cancer metastasis. It is still unknown how varying periods of anchorage loss contribute to anoikis resistance, cell migration, and metabolic reprogramming of cancerous cells.

**Results:**

Our study demonstrated that prolonging the anchorage-free lifetime of non-small-cell lung cancer NCI-H460 cells for 7 days strengthened anoikis resistance, as shown by higher half-life and capability to survive and grow without anchorage, compared to wild-type cells or those losing anchorage for 3 days. While the prolonged anchorage-free lifetime was responsible for the increased aggressive feature of survival cells to perform rapid 3-dimensional migration during the first 3 h of a transwell assay, no significant influence was observed with 2-dimensional surface migration detected at 12 and 24 h by a wound-healing method. Metabolomics analysis revealed significant alteration in the intracellular levels of six (oxalic acid, cholesterol, 1-ethylpyrrolidine, 1-(3-methylbutyl)-2,3,4,6-tetramethylbenzene, β-alanine, and putrescine) among all 37 identified metabolites during 7 days without anchorage. Based on significance values, enrichment ratios, and impact scores of all metabolites and their associated pathways, three principal metabolic activities (non-standard amino acid metabolism, cell membrane biosynthesis, and oxidative stress response) offered potential links with anoikis resistance.

**Conclusions:**

These findings further our insights into the evolution of anoikis resistance in lung cancer cells and identify promising biomarkers for early lung cancer diagnosis.

## Background

Stimuli to trigger cell death can be intracellular or extracellular cues or events, such as cytoskeleton disruption, DNA damage, hypoxia, inflammation, or infection [1–4]. These triggers execute signal transduction cascades responsible for various forms of cell death, varying from apoptotic to necrotic morphotypes [3, 4]. Among the paths toward cell death, anoikis (homelessness) is the predominant pathway when cells lose integrin-mediated cell-to-extracellular matrix interactions [2, 4]. These anchorage-independent cells can be either together or individuals without cadherin-mediated cell-to-cell interactions [5–7]. The molecular mechanisms underlying anoikis have been proposed to be comparable to those described for both intrinsic and extrinsic pathways of apoptosis. Nonetheless, it is still unclear which death receptor and death ligand trigger anoikis through the extrinsic pathway. Hitherto, the activation of caspase-3 (an executioner caspase) via the interplay between mitochondrial outer membrane integrity and the B cell lymphoma-2 (BCL-2) family is the leading cause of anoikis [2, 4], which is in common with the intrinsic pathway of apoptosis.

Adherently growing normal cells are expected to die shortly after losing their anchorage through anoikis, unlike those malignant ones that can develop anoikis resistance. Cancerous cells adopt various strategies to evade anoikis, for instance, anti-apoptotic BCL-2 protein activation, adhesion-related gene silence, focal adhesion kinase signaling perturbation, epithelial-to-mesenchymal transition induction, and others [4]. These anoikis-resistant mechanisms are considered a critical initiator of cancer metastasis – a significant drawback in cancer therapy [8–10]. Cancer metastasis is a multistep cascade whereby malignant cells detach from their primary sites, intravasate and migrate along the vascular and lymphatic systems, and extravasate to form secondary tumors elsewhere in the body [8–10]. Along with these metastatic steps, cancerous cells acquire a set of adaptations, including anoikis resistance, to survive and proliferate under anchorage-free and shear-stressed conditions, avoid immune cell attack, and establish malignant growth in the foreign environment. Hence, proving how varying anchorage-free periods contribute to anoikis resistance in malignant cells may offer a new way to enhance cancer cell death and prevent metastasis.

Among all carcinomas, lung cancer, the world’s most prevalent cancer, threatening human life and causing millions of deaths annually, has been recognized for its high risk of brain, bone, liver, and adrenal gland metastases [10–15]. Based on the histological classification, there are two major subtypes of lung cancer, including non-small-cell lung cancer (NSCLC) – the most prevalent subtype and its less-found counterpart – small-cell lung cancer (SCLC). Approximately nine out of ten lung cancer patients are diagnosed with NSCLC [13, 14]. Although NSCLC is considered less aggressive and has slower progress in metastatic malignancy than SCLC, it does not always respond to chemotherapy because of its higher histopathological variations and molecular distinctions [14, 15]. Since almost no symptoms appear in the early stages of lung cancer, diagnosis is often delayed and metastasis may be underway, leading to the low survival rate of patients with this disease [16]. To improve advanced diagnosis with a hope of preventing metastasis in lung cancer patients, it is essential to understand how lung cancer cells evolve anoikis resistance after losing their anchorage dependency.

Some clinical research attempts to optimize lung cancer diagnosis by searching for the metabolites associated with the disease incidence, using state-of-the-art facilities in molecular biology [16–21]. Over 2000 metabolites have been profiled in various specimens (e.g., sera, plasma, and tissues) derived from lung cancer patients [21], while very few of them are identified statistically as lung cancer biomarkers [16–21]. To date, over 600 metabolites remain unknown for their classification and roles in lung cancer metabolism [21]. Only a few studies unveil the metabolic alterations associated with anoikis resistance, such as elevations in glycolysis, asparagine bioavailability, and fatty acid uptake [22]. With a focus on lung cancer, the increased level of glutamate dehydrogenase 1 (GDH1) shows a significant link to anoikis resistance and metastasis initiation in serine-threonine kinase (LKB1)-deficient NSCLC cells [23]. In addition, the reduced ratio of phosphoribosyl pyrophosphate amidotransferase (PPAT) per glutaminase (GLS1) in glutamine nitrogen metabolism exerts suppressive roles in SCLC cell growth and metastasis [24, 25]. More studies are yet required to fill the remaining knowledge gaps on how lung cancer cells maintain their metabolisms to defeat anoikis.

In this study, we assessed how varying periods of being without anchorage of NSCLC NCI-H460 cells contribute to their anoikis resistance, metastatic features, and metabolic activity. A set of in vitro assays was conducted to investigate the impact of anchorage-free duration on cell survival, growth, and migration. A gas chromatography-mass spectrometry (GC-MS)-based metabolomic analysis was also employed to elucidate differential metabolites and their associated metabolic pathways that cells evolved to resist anoikis.

## Results

### Prolonged anchorage independence strengthens anoikis resistance and early migration of NSCLC cells

**Fig. 1:**
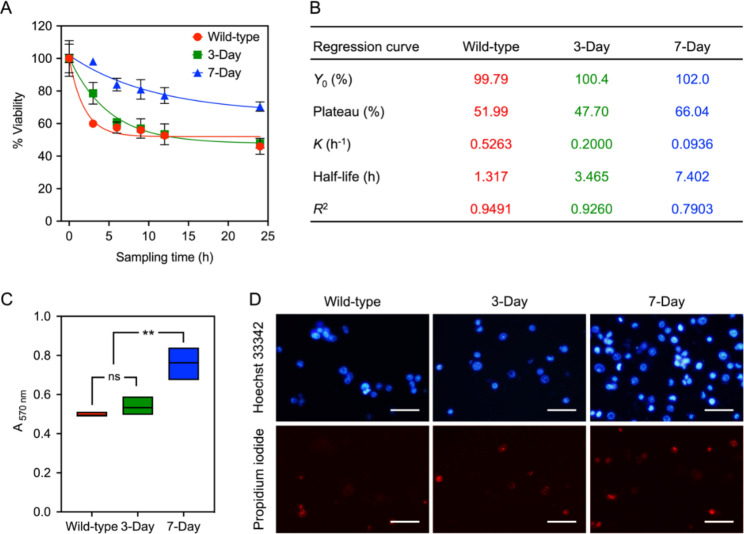
Prolonged anchorage-free lifetimes of NCI-H460 cells enhance their survival, proven by a 3-(4,5-dimethylthiazol-2-yl)-2,5-diphenyltetrazolium bromide (MTT)-based cell viability assay. Either 3- and 7-day-induced anoikis-resistant or wild-type cells were assessed for their capability to survive in low-nutrient anchorage-free conditions and recover their adherent growth ability. **(A)** The percentage of cell viability was computed and reported as a relative percentage value to that measured at time zero. **(B)** Regression curves generated from the plots between cell viability percentages and sampling times in A were mathematically interpreted, where *Y*_0_ = y-intercept, *K* = slope, and *R*^2^ = coefficient of determination. **(C)** The absorbance values for cell samples collected at time zero and measured by the MTT-based cell viability assay after allowing cell attachment for up to 27 h, were reported, where ns = not significant and ** = *p* < 0.01. **(D)** A set of representative views demonstrating dead cells (stained with both bright blue of Hoechst 33342 and red of propidium iodide) after being without anchorage for 24 h assessed by a dual nuclear staining method, was randomly selected, where scale bars = 5 μm. All graphical results represent means ± standard deviations derived from at least triplicate experiments

Survival NCI-H460 cells from undergone low-nutrient anchorage-free conditions for 3 and 7 days were sorted and assessed for their anoikis-resistant capability compared to their wild-type cells (adherently growing under typical conditions) (Fig. 1). These cells were allowed to be in low-nutrient anchorage-free conditions once again for up to 24 h and collected at varying time points to track the level of their anoikis resistance estimated by the viability of adherent cells. Regardless of cell types tested, the proportion of adherently viable cells gradually dropped when we extended their anchorage-free durations (Fig. 1A). However, the declining rates of cell viability (*K*) varied from the lowest (0.0936 h^− 1^) for 7-day-induced anoikis-resistant cells to the highest (0.5263 h^− 1^) for wild-type controls and were likely to plateau after 12 h of being without anchorage (Fig. 1A, B). With the extra sum-of-squares *F* test, the difference among the declining rates of cell viability was significant (*p* < 0.0005), and applying y-intercepts, plateaus, and the declining rates into the test indicated that all regression curves were significantly different (*p* < 0.0001) (Fig. 1A, B).

NCI-H460 cells induced to resist anoikis for 7 days (66.04%) had around 14–18% higher plateau than that of 3-day-induced anoikis-resistant (47.70%) and wild-type (51.99) ones (Fig. 1A, B). This result was positively correlated with the density of adherently viable cells sampled at time zero of losing anchorage and allowed to attach to the surface for 27 h (Fig. 1C) and was also positively correlated with the density of viable cells collected and assessed after being without anchorage for 24 h (Fig. 1D). The capability of NCI-H460 cells exposed to the low-nutrient medium for 27 h (without physical perturbations) to survive and recover their anchorage dependence was similar between wild-type controls and those induced to resist anoikis for 3 days but was nearly 1.5-fold less than that of 7-day-induced anoikis-resistant cells (Fig. 1C).

**Fig. 2:**
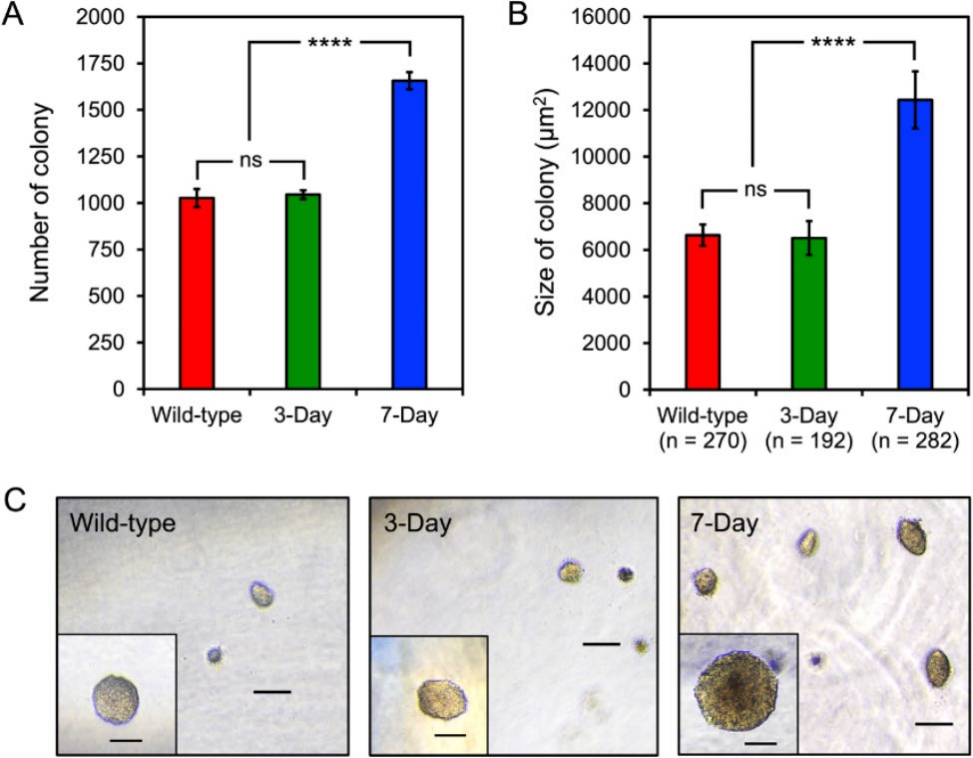
Prolonged anchorage-free lifetimes of NCI-H460 cells strengthen their survival and growth as shown by a soft agar colony formation assay. Either 3- and 7-day-induced anoikis-resistant or wild-type cells were assessed for their capability to survive and grow under the anchorage independence conditions. The number **(A)** and size **(B)** of colonies formed in the soft agar medium was measured, where ns = not significant, *** = *p* < 0.0001, and n = number of colonies assessed. **(C)** A set of representative views demonstrating the variation in density and size of colonies survived and grown in the soft agar medium for 7 days, was randomly selected, where scale bars = 100 μm and 50 μm (for the close-up views). All graphical results represent means ± standard errors of means derived from at least triplicate experiments

Based on our cell viability assessment, it was obvious that prolonged anchorage-free survival could strengthen anoikis resistance, since cells without anchorage for 7 days had the highest half-life (7.402 h) followed by 3-day-induced anoikis-resistant (3.465 h) and wild-type (1.317 h) cells (Fig. 1B). To confirm the capability of different NCI-H460 cells to survive and grow under anchorage-free conditions, a soft agar colony formation assay was conducted (Fig. 2). Significantly higher numbers (Fig. 2A) and significantly larger sizes (Fig. 2B, C) of colonies formed in the soft agar proved that cells induced to resist anoikis for 7 days could maintain their anchorage-independent survival to a greater extent than other NCI-H460 cells tested.

**Fig. 3:**
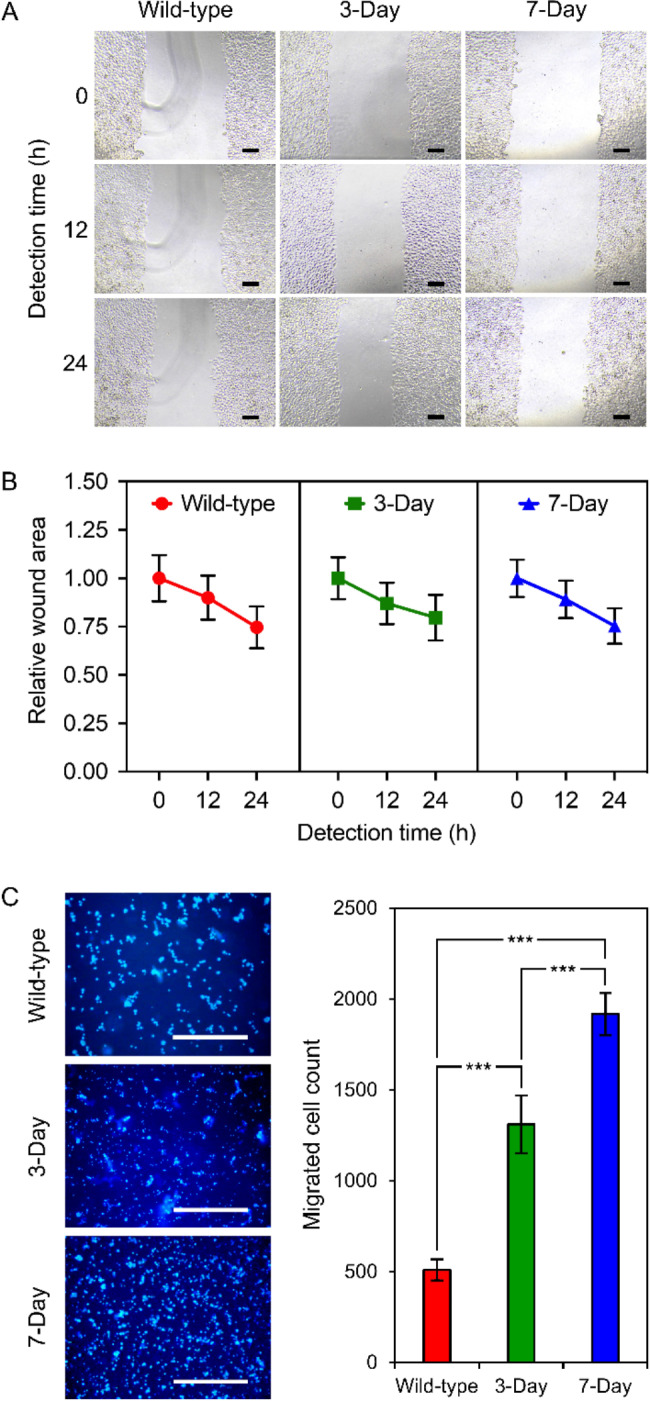
Prolonged anchorage-free lifetimes of NCI-H460 cells do not significantly influence 2-dimensional collective migration but promote early 3-dimensional individual migration. Either 3- and 7-day-induced anoikis-resistant or wild-type cells were assessed for their capability to migrate collectively for up to 24 h and individually for 3 h by a wound-healing assay (A and B) and a transwell migration method (C), respectively. **(A)** A set of representative views demonstrating the variation in wound areas measured at 0, 12, and 24 h of the incubation, where scale bars = 100 μm. **(B)** The change in wound areas was computed and reported as a relative wound area to that detected at time zero. **(C)** A set of representative views (scale bars = 100 μm) showing cells migrated to the bottom part of the transwell membrane after staining with Hoechst 33342 and the average migrated cell counts, where *** = *p* < 0.0005. All graphical results represent means ± standard deviations derived from at least triplicate experiments

We also assessed how varying anchorage-free lifetimes affected 2- and 3-dimensional migration of NCI-H460 cells (Fig. 3). Two-dimensional cell migration to prove surface migration of adherent cells was conducted using a wound-healing assay (Fig. 3A and B), which unveiled no significant difference in wound areas covered by different cells tested at 12 and 24 h (Fig. 3A). The migration rates derived from the curves between relative wound area and detection times (Fig. 3B) also showed no significant difference. However, 3-dimensional cell migration tested by a transwell approach demonstrated significant difference (*p* < 0.0005) in the number of cells migrated during the first 3 h of being without anchorage (Fig. 3C). It was obvious that cells formerly lost anchorage for 7 days exhibited fastest relocation proven by the highest number of migrated cells (1918 ± 116 cells), followed by 3-day-induced anoikis-resistant (1311 ± 159 cells) and wild-type cells (510 ± 58 cells), respectively (Fig. 3C).

**Metabolites associated with non-standard amino acid metabolism, cell membrane biosynthesis, and antioxidant activity exhibit potential links to anoikis resistance of NSCLC cells**.

**Fig. 4:**
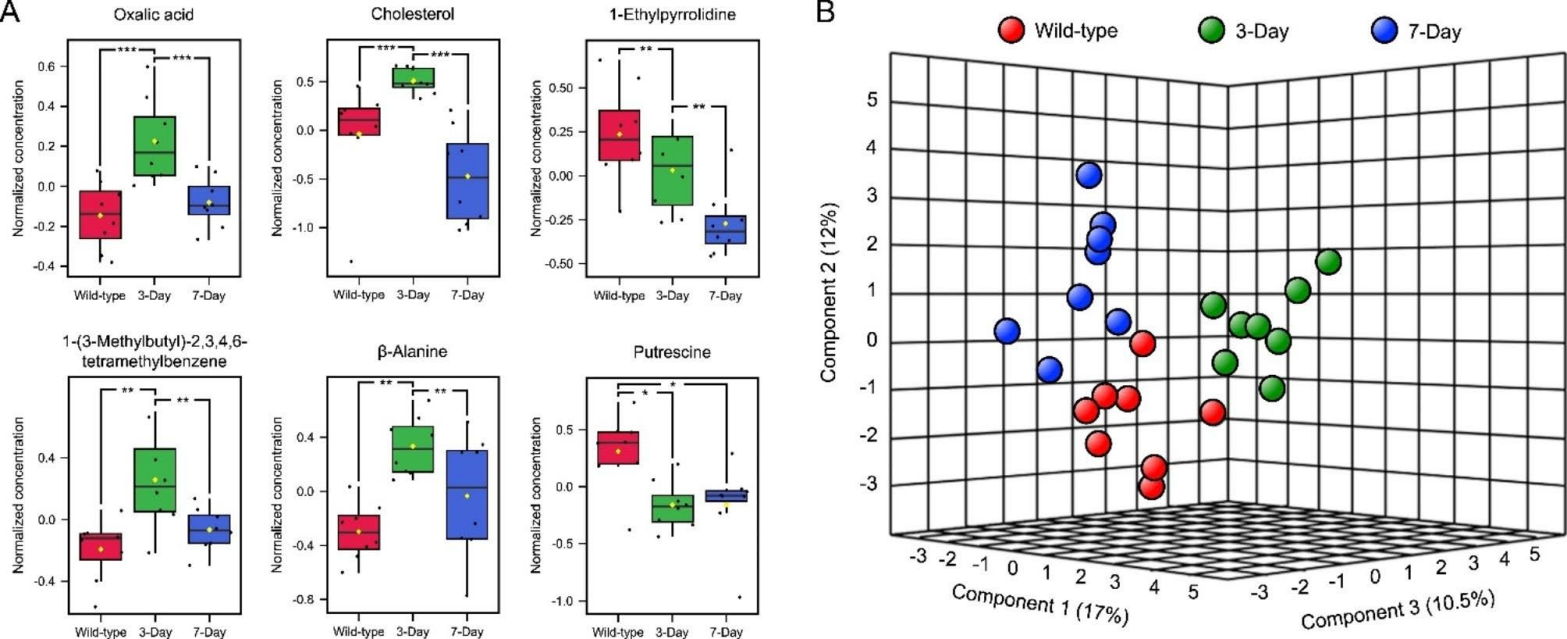
Boxplots of significantly differential metabolites and sample clustering by sparse partial-least-squares discriminant analysis of metabolite profiles detected in 3- and 7-day-induced anoikis-resistant or wild-type NCI-H460 cells. **(A)** The significantly differential metabolites were determined based on their normalized concentrations, where * = *p* < 0.01, ** = *p* < 0.005, and *** = *p* < 0.001. (B) This 3-dimensional clustering view represents the similarities and dissimilarities between eight metabolite profiles per each cell type

A total of 37 metabolites found across different NCI-H460 cells tested were identified by a GC-MS-based metabolomics approach (Table 1). Six metabolites (oxalic acid, cholesterol, 1-ethylpyrrolidine, 1-(3-methylbutyl)-2,3,4,6-tetramethylbenzene, β-alanine, and putrescine) showed significant difference (*p* < 0.05) in their levels across all cell types (Table 1; Fig. 4A). Most of these metabolites (i.e., oxalic acid, cholesterol, 1-(3-methylbutyl)-2,3,4,6-tetramethylbenzene, and β-alanine) were significantly increased in cells losing anchorage for 3 days, and significantly decreased in those being without anchorage for 7 days, down to the same level as wild-type controls (Fig. 4A). Only 1-ethylpyrrolidine had a consistent decline in its intracellular level in a time-dependent manner, from levels similar to those in wild-type controls down to levels observed in 3- and 7-day-induced anoikis-resistant cells, respectively (Fig. 4). For the intracellular levels of putrescine, this metabolite significantly decreased in cells without anchorage for 3 or 7 days, compared to wild-type controls (Fig. 4A). Most of the decline in putrescine levels occurred during the first 3 days without anchorage and was then relatively constant through day 7. Using supervised classification by a multilevel sparse partial-least-squares discriminant analysis (sPLS-DA) of metabolite profiles across different NCI-H460 cells tested in this study, three clusters were observed in the fittest distribution plot (Fig. 4B). Each cluster referred to the similarity of metabolite profiles derived from the same cell type. Considering the correlation across different cell types, the metabolite profiles of cells being without anchorage for 7 days were closer to those of wild-type controls than those of 3-day-induced anoikis resistant cells (Fig. 4B). This observation was consistent with the levels of many significantly differential metabolites (e.g., oxalic acid, cholesterol, 1-(3-methylbutyl)-2,3,4,6-tetramethylbenzene, and β-alanine) found across different cell types (Fig. 4A).

**Table 1:** Metabolites identified across 3- or 7-day-induced anoikis-resistant and wild-type NCI-H460 cells

Retention time (min)	Metabolites	Identification	*p*-value
4.013	O-Ethylhydroxylamine	MS, RI	ns
4.853	Octane	MS, RI	ns
7.564	1-(3-Methylbutyl)-2,3,4,6-tetramethylbenzene	MS, RI	< 0.05
7.958	Hydroxylamine	MS, RI	ns
8.614	1-Piperidinecarboxaldehyde	MS, RI	ns
8.629	1-Ethylpyrrolidine	MS, RI	< 0.05
8.864	Oxalic acid	MS, RI	< 0.001
8.984	Isopropyl propionate	MS, RI	ns
9.093	N-methylmetadrenaline	MS, RI	ns
10.582	Dodecane	MS, RI	ns
11.982	Tyramine	MS, RI	ns
12.005	Ethanolamine	MS, RI	ns
12.424	Diethylene glycol	MS, RI	ns
12.429	Benzene, 1,3-bis(1,1-dimethylethyl)-	MS, RI	ns
12.548	Carbamic acid	MS, RI	ns
13.602	Phosphate	MS, RI	ns
13.723	Glycerol	MS, RI	ns
13.915	Phosphonic acid derivative	MS, RI	ns
13.967	Tridecane	MS, RI	ns
14.553	Glycine	MS, RI	ns
14.770	1-Decanol	MS, RI	ns
15.055	Cyclohexasiloxane, dodecamethyl-	MS, RI	ns
18.509	β-Alanine	MS, RI	< 0.05
20.734	Malic acid	MS, RI	ns
23.042	Ethyne, fluoro-	MS, RI	ns
23.242	Diethyl Phthalate	MS, RI	ns
24.512	L-Glutamic acid	MS, RI	ns
26.024	Pyrophosphate	MS, RI	ns
27.506	Putrescine	MS, RI	< 0.05
29.024	Phosphorylethanolamine	MS, RI	ns
30.152	Myristic acid	MS, RI	ns
30.922	Phenylephrine	MS, RI	ns
31.771	Alanylglycine	MS, RI	ns
32.375	Glucose	MS, RI	ns
36.736	Myo-Inositol	MS, RI	ns
39.287	Stearic acid	MS, RI	ns
55.701	Cholesterol	MS, RI	< 0.001

The significance (*p*)-value among different cell types for each metabolite was determined by analysis of variance, where ns = not significant (*p* > 0.05); MS = mass spectra, RI = retention index

**Fig. 5:**
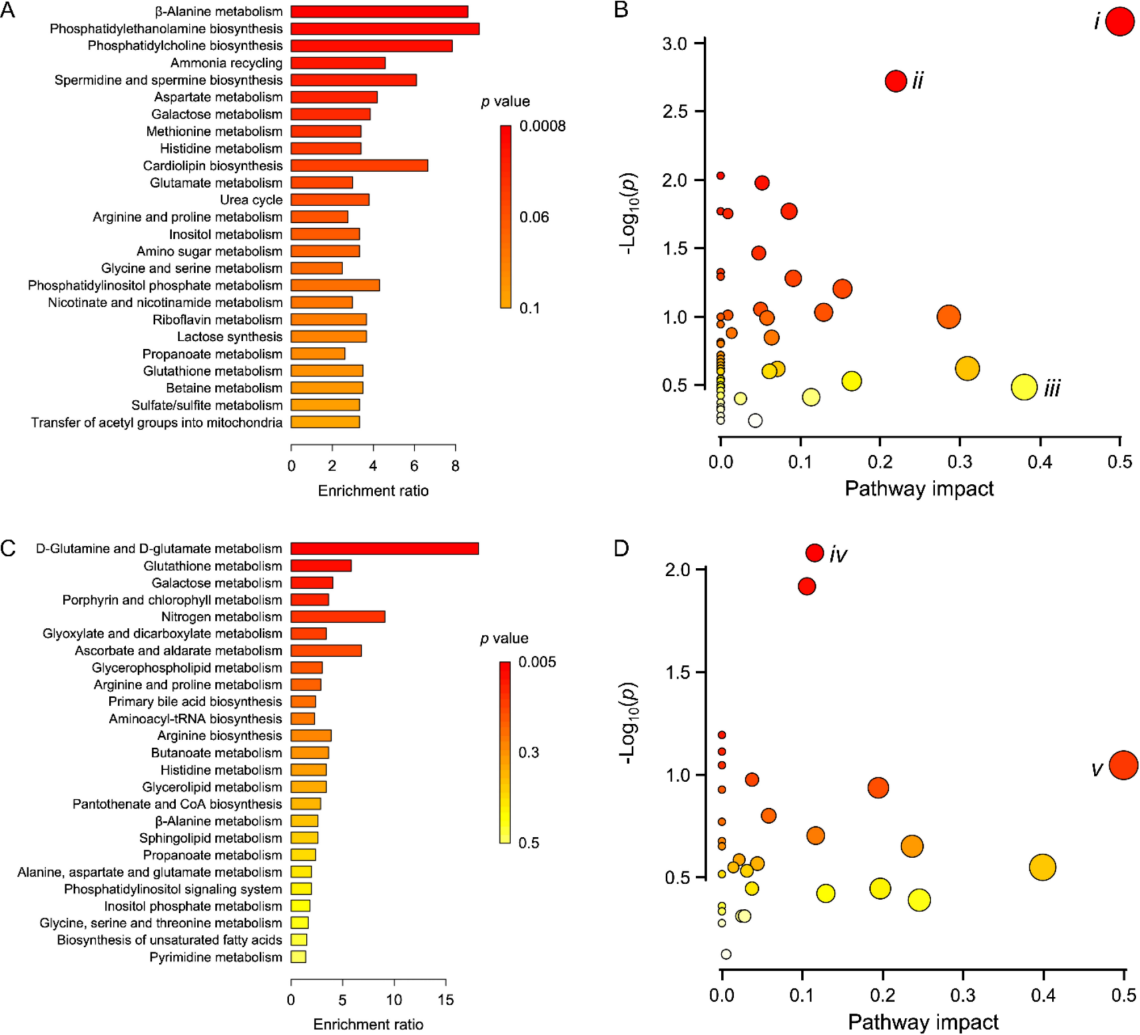
Top 25 altered metabolic pathways and scatter plots showing the impacted metabolic pathways in 3- and 7-day-induced anoikis-resistant or wild-type NCI-H460 cells. These pathways were identified and annotated by comparisons with two databases: the Small Molecule Pathway Database **(A and B)** and the Kyoto Encyclopedia of Genes and Genomes **(C and D)**. The top 25 altered metabolic pathways were ranked based on the significance (*p*) values and the enrichment ratios **(A and C)**. The scatter plots were constructed based on the significance (*p*) value (color scale) and impact score (plot size) of each pathway **(B and D)**. Some plots that associated with the top ranked pathways and having relatively high *p* values, enrichment ratios, and impact scores, were determined, including *i* = phosphatidylethanolamine biosynthesis, *ii* = phosphatidylcholine biosynthesis, *iii* = β-alanine metabolism, *iv* = glutathione metabolism, and *v* = D-glutamine and D-glutamate metabolism (B and D)

Each identified metabolite was incorporated into its associated metabolic pathway determined by comparing with the Small Molecule Pathway Database (SMPDB) (Fig. 5A and B) and the Kyoto Encyclopedia of Genes and Genomes (KEGG) (Fig. 5C and D). Top 25 associated pathways with varying enrichment ratios and significance (*p*) values were listed (Fig. 5A and C). With SMPDB (Fig. 5A), associated pathways were ranked with greater *p* values (0.0008–0.1) but lower enrichment ratios (< 10) than those ranked with KEGG (Fig. 5C), where *p* values were 0.005–0.5 and enrichment ratios were < 20. These ranking lists were comparable to what was observed with the scatter plots between *p* values and impact scores of the associated pathways (Fig. 5B and D). Based on *p* values, enrichment ratios, and impact scores, five metabolic pathways (β-alanine metabolism, phosphatidylethanolamine biosynthesis, phosphatidylcholine biosynthesis, D-glutamine and D-glutamate metabolism, and glutathione metabolism) were determined to have potential links with the development of anoikis resistance in NCI-H460 cells (Fig. 5). The pathways were categorized into three principal metabolic activities, including non-standard amino acid metabolism (β-alanine, D-glutamine, and D-glutamate), cell membrane biosynthesis (phosphatidylethanolamine and phosphatidylcholine), and oxidative stress response (glutathione metabolism).

## Discussion

The knowledge on how varying anchorage-free lifetimes of malignant cells contribute to their survival, growth, and migration is scarce. These fundamental insights strongly link to the evolution of anoikis resistance and the initiation of metastasis, which are essential for the future development of cancer therapy. Some studies demonstrated that NCI-H460 cell viability gradually decreased from 100% at time zero to ~ 30% [26] or ~ 60% [27] at 24 h of losing anchorage. Our study found the plateau at 51.99% for the wild-type NCI-H460 cells (Fig. 1A, B) was in the range mentioned above. This variation in the extent of decline of NCI-H460 cell viability might be due to the difference in cell ages, medium composition, nutrient levels, and physical inducers used in the various experiments. It was highly possible that nutrient scarcity at the early phase of being without anchorage was responsible for the limited survival of NSCLC cells. Cells detaching from their matrix often face several cellular stressors before undergoing anoikis, typically oxidative stress, nutrient depletion, and energy shortage [28–34]. Many malignant cells can modify their metabolic activity to survive anoikis under such stressed conditions. For instance, some cancerous cells can adopt autophagy (a self-eating process) as a short-term anoikis resistance strategy to maintain anchorage-free survival [28–30], while many others can reprogram their metabolism from anabolism to catabolism through AMP-activated protein kinase (AMPK)-mediated cellular homeostasis [31–34]. Additional studies are essential to understand how varying nutrient levels (nutrient bioavailability) contribute to anoikis and the switch between anchorage-dependent and anchorage-independent survival of malignant cells.

Cancerous cell relocation is among metastatic features used to define the aggressiveness of malignant cells. The assessment of cell migration by a wound-healing approach relies on collective migration (based on cell-to-cell interaction), which requires at least an 80% confluent cell monolayer in a nutrient-rich circumstance before the test [35]. With these criteria in the experimental set-up, cells formerly induced to survive from any anchorage-free periods might have already recovered their adherent growth ability and collective migration capabilities. Moreover, the migration rate derived from this experiment relied on 2-dimensional wound areas computed after 24 h of incubation. That is why we could not observe the significant effect of varying anchorage-free lifetimes formerly undergone. Other cell migration assessments [35], e.g., individual cell migration tests, may offer further insight into the impacts of anchorage independence and nutrient deprivation on the migration capability of malignant cells with varying anoikis resistance strength. An additional study based on the 3-dimensional relocation of individual cells using a transwell assay unveiled that prolonging anchorage-free lifetimes of NCI-H460 cells could provoke their migration capability. However, it was noticeable that the significant effect was only observed at the early phase (3 h) of the test, not at 7 h (data not shown).

Since prolonging anchorage-free lifetimes of NCI-H460 cells could enhance their anoikis resistance and early migration, we investigated further how these characteristics linked with cellular metabolisms. Although only 37 metabolites were found across different NCI-H460 cells, we are unsurprised as an untargeted metabolomic analysis using a GC-MS platform was employed to profile metabolites in only one cell lineage. Some studies applied GC-MS to profile metabolites in various and complex specimens (e.g., lung tissues, sera, and bronchoalveolar lavage fluids) collected from lung cancer patients but still found less than 100 total or significantly differential metabolites [36–39]. Among all metabolites found in our study, six (oxalic acid, cholesterol, 1-ethylpyrrolidine, 1-(3-methylbutyl)-2,3,4,6-tetramethylbenzene, β-alanine, and putrescine) were significantly different when prolonging anchorage-free lifetimes of NCI-H460 cells. Oxalic acid is commonly known as oxalate (a conjugate base of oxalic acid), involved in some metabolic pathways, e.g., purine metabolism. It was found to be significantly increased in lung tissues [36] and sera [40] derived from lung cancer patients regardless of their smoking backgrounds, which was contrary to another study that reported a significant downregulation of this metabolite in blood sera of lung cancer patients compared to those of healthy volunteers [37]. Our study also found fluctuation in levels of this metabolite in NCI-H460 cells without anchorage for 3 or 7 days (Fig. 4A). Moreover, Hori et al. [36] noted that oxalic acid levels in lung cancer tissues shifted on a diurnal basis. With these findings, the developmental phases of the disease and cells, along with sample type (cells, tissues, and sera) may affect the detection levels of oxalic acid. These factors should be considered prior to its’ designation as a cancer biomarker.

Cholesterol – a macromolecular lipid and an essential building block of mammalian cell membranes, exhibited similar dynamics to oxalic acid (Fig. 4A). Many studies found that cholesterol levels inversely correlated with the progression of lung cancer, as diverse tissues from diseased patients had significantly lower levels of cholesterol than those of non-diseased volunteers [38–40]. This cholesterol depletion was proposed to have a potential link with lung cancer cell proliferation through the interplays between this metabolite, Ca^2+^, and calcium/calmodulin-dependent protein kinase type 1D [39]. Metabolomics data regarding 1-ethylpyrrolidine and 1-(3-methylbutyl)-2,3,4,6-tetramethylbenzene are scarce. While *N*-oxide-derivatized ethylpyrrolidine of an Src kinase inhibitor (an anti-oncogenesis molecule) was proved to be one of the predominant metabolites found in human, dog, and rat [41], 1-(3-methylbutyl)-2,3,4,6-tetramethylbenzene was found to be downregulated in patients with gingivitis compared to healthy controls [42]. Both were reported for the first time in this study to associate with NSCLC. Their intracellular levels were altered differently, as 1-ethylpyrrolidine gradually decreased during anchorage-free periods, but 1-(3-methylbutyl)-2,3,4,6-tetramethylbenzene exhibited an up-down pattern (Fig. 4A). The 1-ethylpyrrolidine depletion seems to support its role as a part of the Src kinase inhibitor to promote cancer progression [43]. Additional studies are needed to perceive how these metabolites contribute to lung cancer cell metabolism and anoikis resistance.

Increased serum levels of β-alanine are among the recognized characteristics of NSCLC patients [44]. A study focusing on the nucleotide metabolism in human lung cancer tissues revealed that the tissue levels of β-alanine as a catabolite from pyrimidine metabolism were elevated compared to the non-diseased controls [45]. Our study also found a significant increase of β-alanine after inducing NCI-H460 cells to resist anoikis for 3 days, following by its significant decrease on day 7th (Fig. 4A). Another significantly differential metabolite found in our study was putrescine, which significantly dropped for its intracellular levels after being without anchorage for 3 and 7 days compared to the wild-type controls (Fig. 4A). While Moreno et al. [45] found significantly high levels of putrescine in lung tumor tissues compared to those in non-malignant ones, Wikoff et al. [46] observed unchanged levels of putrescine in similar samples. These inconsistent findings are not unusual. In addition to the variations in sample classification, measuring a metabolite (a highly dynamic player in one or more metabolic pathways) at a single time point without awareness of spatiotemporal influences may not be sufficient to interpret its alteration.

It was obvious that malignant cells advantageously reprogramed their metabolic modes from anabolism to catabolism when they faced anchorage independence stresses, which was compatible with what described by Herzig and Shaw [34]. The catabolic center was switched from usual to unusual pathways, where β-alanine and putrescine act as centric metabolites to reduce energy consumption (e.g., purine metabolism), maintain cell proliferation (e.g., D-amino acid metabolism and glycerophospholipid metabolism), and defeat oxidative stress (glutathione metabolism). The crosstalk between purine metabolism and mitochondrial function with regard to energy supply in cancer cells has been previously summarized [47], while another study unveiled the roles of D-amino acids in promoting cancer cell proliferation [48].

## Conclusions

NSCLC NCI-H460 cells losing anchorage for 7 days showed greater anoikis resistance strength than those without anchorage for 3 days or wild-type controls, proven by the capability to survive and grow under the anchorage-free conditions. A metabolomics study revealed 37 metabolites across different cells tested, while six (oxalic acid, cholesterol, 1-ethylpyrrolidine, 1-(3-methylbutyl)-2,3,4,6-tetramethylbenzene, β-alanine, and putrescine) were significantly different based on their detectable concentrations. With all variations in the elucidated metabolite profiles, three associated metabolic pathways (i.e., non-standard amino acid metabolisms, cell membrane biosynthesis, and antioxidant activity) were proposed to have potential links with the anoikis resistance strength of NCI-H460 cells.

## Methods

### Culture medium, chemicals, and reagents

The Roswell Park Memorial Institute (RPMI) 1640 medium, fetal bovine serum (FBS), penicillin/streptomycin solution, phosphate-buffered saline (PBS) pH 7.4, L-glutamine, and trypsin, were bought from Gibco™ (Gaithersburg, MA, USA). Other chemicals, i.e., 3-(4,5-dimethylthiazol-2-yl)-2,5-diphenyl tetrazolium bromide (MTT), dimethyl sulfoxide (DMSO), Hoechst 33342, propidium iodide (PI), agarose powder, acetone [HPLC grade], ethanol [HPLC grade], methoxyamine hydrochloride (MeOX), pyridine, *N*-methyl-*N*-(trimethylsilyl)-trifluoroacetamide (MSTFA), and trimethylchlorosilane (TMCS), were purchased from Sigma-Aldrich (St. Louis, MO, USA).

### Cell culture and growth conditions

Human NSCLC NCI-H460 cells were ordered from the American Type Culture Collection (Manassas, VA, USA). NCI-H460 cells were grown as monolayers in 10% (v/v) FBS-supplemented complete RPMI medium (containing 2 mM L-glutamine and 100 units mL^− 1^ penicillin/streptomycin solution) at 37 °C in a humidified incubator with 5% CO_2_ saturation. When reaching ~80% confluence, cells were subjected to sub-culturable cycle by trypsinization using 0.25% trypsin.

### Preparation of anoikis-resistant cells

To examine how prolonged anchorage-free lifetime of NCI-H460 cells affect their anoikis resistance, metastatic features, and metabolic activity, cells were induced to resist anoikis by exposing them to low-nutrient anchorage-free conditions for 3 or 7 days. Cells grown as monolayers as mentioned previously were collected after trypsinization into 1.5 mL tubes by centrifugation at 1118* g* and 4 °C for 5 min. Collected cells were resuspended in 1% (v/v) FBS-supplemented complete RPMI medium at a density of 1.5 × 10^5^ cells mL^− 1^ and seeded in an ultra-low attachment 6-well plate. The seeded plate was incubated in a humidified incubator at 37 °C with 5% CO_2_ for 3 and 7 days. During the incubation, cells were separated to prevent cells from anchoring to each other by pipetting ten times a day. When reaching the incubation time, all cells were collected by centrifugation at 1118* g* and 4 °C for 5 min. Collected cells were resuspended in 10% (v/v) FBS-supplemented complete RPMI medium, seeded in a culture plate, and incubated at 37 °C in a humidified incubator for 2 days. After one day of incubation, the medium was removed, only the remaining adherent cells were washed once with PBS pH 7.4, and fresh medium was added. These cells were collected by trypsinization after day 2 of incubation and determined as 3- or 7-day-induced anoikis-resistant cells.

### Assessment of anoikis resistance strength

Three-day and 7-day-induced anoikis-resistant or wild-type (no anoikis-resistant induction) NCI-H460 cells were used to examine the effects of prolonged anchorage-free lifetime on resistance to anoikis. The strength of anoikis resistance was estimated by the capability of cells to survive and grow under low-nutrient anchorage-free conditions, using a set of in vitro assays. Prior to the tests, cells at a density of 1.5 × 10^5^ cells mL^− 1^ were subjected to be without anchorage by suspending in 1% (v/v) FBS-supplemented complete RPMI medium, which was seeded in an ultra-low attachment 6-well plate and incubated at 37 °C in a humidified incubator for 24 h.

A modified MTT cell viability assay was carried out to assess the capability of cells to survive from anoikis and recover their adherent growth ability. At 0, 3, 6, 9, 12, and 24 h of incubation, every cell suspension prepared above was mixed by pipetting and sampled (100 µL) to fill in a 96-well plate. At 3 h after the last sampling, the medium in each well was removed and replaced with 100 µL of 0.5 mg mL^− 1^ MTT solution. The assayed plate was incubated in the dark for 3 h at 37 °C in a humidified incubator. Then, the solution was removed and replaced with 100 µL of DMSO to dissolve the formed formazan crystal, which was measured with the absorbance at 570 nm wavelength using a microplate reader (Anthros, Durham, NC, USA). The absorbance values measured at each sampling point relative to those detected at time zero were computed as the percentages of cell viability. The difference in absorbance values assessed for the 0 h of sampling time was also denoted.

Viable and dead cells after being without anchorage for 24 h were confirmed with a dual nuclear staining assay. The cell suspension (500 µL) left from the above experiment was transferred into 1.5 mL tube, and cells were collected by centrifugation at 1118* g* and 4 °C for 5 min. Collected cells were resuspended in 100 µL of PBS pH 7.4 and stained with 2 µg mL^− 1^ Hoechst 33342 and 1 µg mL^− 1^ PI, which was incubated in the dark for 30 min at 37 °C in a humidified incubator. Stained cells were observed and discriminated under a fluorescence microscope (Olympus IX51 with DP70, Tokyo, Japan), while those with condensed and/or fragmented nuclei and emitted both bright blue (Hoechst 33342) and red (PI) fluorescence were considered dead cells.

A modified soft agar colony formation technique was established to ensure the capability of cells to survive and grow under anchorage-free conditions. Cells being without anchorage for 24 h, as previously prepared, were used in this experiment. A double-layer agar was conducted in a 24-well plate, where the bottom (500 µL) and upper (250 µL) layers contained 0.5% and 0.3% (w/v) agarose, respectively. Both layers were made by diluting 1.5% (w/v) agarose gel suspension with 10% (v/v) FBS-supplemented complete RPMI medium, and the upper layer contained 2 × 10^3^ cells. After setting for 3 h, the medium (250 µL) was added into each well and re-filled every third day of the incubation. The assayed plate was incubated at 37 °C in the humidified incubator for 7 days. All colonies formed were counted and randomly photographed on day 7th of incubation under a camera-equipped microscope (Nikon Ts2, Tokyo, Japan). The colony size (area) was measured using ImageJ (https://imagej.nih.gov/ij/). The number and size of colonies are measures of the capability of cells to survive and grow without anchorage, respectively.

### Evaluation of cell migration

The migration ability of cancerous cells is one of the metastatic features indicating their capability to invade into a secondary tumor site. We determined if 3- and 7-day-induced anoikis-resistant NCI-H460 cells could migrate differently on the surface compared to their wild-type siblings using a modified wound-healing assay [35]. Cells were collected from their monolayer cultures and resuspended in 10% (v/v) FBS-supplemented complete RPMI medium. Each cell suspension was seeded in a 96-well plate at a density of 6 × 10^5^ cells mL^− 1^ and incubated overnight at 37 °C in a humidified incubator. A wound as a vertical line on the cell monolayer was generated at the center of the well by scraping with a 20 µL pipette tip. The medium containing cell debris was removed and replaced with fresh medium. The assayed plate was continuously incubated at the same conditions up to 24 h. At 0, 12, and 24 h of the incubation, the wound area was visualized and photographed under the camera-equipped microscope and measured using ImageJ. The 2-dimensional cell migration rates were computed from the slopes of regression curves plotted between wound areas and detection times.

NCI-H460 cells that survived from varying anchorage-free durations were tested further for their 3-dimensional migration using a Boyden chamber 24-well microplate (Corning Costar, State, MA, USA) as previously described with some modifications [49]. Either 3- and 7-day-induced anoikis-resistant or wild-type cells growing as a monolayer in 10% (v/v) FBS-supplemented complete RPMI medium were collected and resuspended in 1% (v/v) FBS-supplemented complete RPMI medium. This cell suspension (100 µL) at a density of 5 × 10^4^ cells per well was seeded on a filter membrane (8 μm pore size) embedded in the transwell insert. Then, each insert was placed in a well of a 24-well microplate containing 500 µL of 10% (v/v) FBS-supplemented complete RPMI medium and incubated for 3 and 7 h. The ability of cells to migrate from the insert into the bottom part of the membrane was examined and photographed under a fluorescence microscope (Olympus IX51, Tokyo, Japan) after staining with 10 µM Hoechst 33342 for 30 min. The migrated cells stained with Hoechst 33342 were counted using ImageJ.

### GC-MS-based metabolome analysis

An untargeted metabolomics analysis was done to identify metabolites and their mediated metabolic pathways altered during the induction of anoikis resistance. Either 3- and 7-day-induced anoikis-resistant or wild-type NCI-H460 cells grown as monolayers were collected and resuspended in 10% (v/v) FBS-supplemented complete RPMI medium (eight replicas per each cell type). Each cell suspension was seeded in a 6-well plate at a density of 1 × 10^6^ cells per well and incubated at 37 °C in the humidified incubator for 12 h. Then, the medium was removed, and metabolites were extracted from adherent cells by quenching in ice-cold 80% (v/v) ethanol with scraping and sonicating at 4 °C in an ice bath for 10 min [50]. The extracts were pooled and centrifuged at 16,000* g* and 4 °C for 3 min, where the supernatants were collected and evaporated by a centrifugal concentrator.

To optimize metabolite separation, detection, and identification by GC-MS, the extracted metabolites were subjected to a two-step derivatization to reduce polarity and increase volatility [51], including (1) methoxymation to turn their enol forms of aldehydes and ketones into oximes or alkyloximes and (2) silylation to introduce silyl groups into their molecules. The first step was carried out by suspending each dried metabolite sample in 100 µL of MeOX solution [20 mg mL^− 1^ of MeOX in pyridine] and vortexing at ambient temperature for 1 h, while the second was performed by saturating each dried metabolite sample in 100 µL of MSTFA plus 1% TMCS and incubating at 70 °C for 30 min. Derivatized samples were then filtered through a 0.22 μm membrane filter and collected in a glass GC vial.

Each metabolite sample was separated by the Agilent 7000 C Triple Quadrupole GC/MS System (Agilent Technologies, Santa Clara, CA, USA) comprised of Agilent 7890B GC with an HP-5 ms column (30 m × 0.25 mm × 0.25 μm) and Agilent 7000 C MS. The derivatized sample (1 µL) was injected using a 10 µL syringe-equipped autosampler with 1:10 split ratio into a straight glass wool liner split, using helium as a carrier gas at a flow rate of 1.0 mL min^− 1^. Acetone was used for rinsing the syringe thrice before every injection. The mass spectrometer transfer line was maintained at 300 °C, while the inlet temperature at 250 °C was set. The oven temperature started at 85 °C for 5 min, climbed up at 8 °C min^− 1^ to hold at 205 °C for 5 min, and increased again at the same rate to hold at 300 °C for 15 min, while the ion source temperature at 230 °C was set. The MS was conducted in an electron impact mode (70 eV) at a rate of 2 scans s^− 1^, and full-scan mass spectra from 33 to 650 m/z were acquired at a rate of 5 spectra s^− 1^. The alkane series mixture (C_10_ to C_40_) was used to standardize the retention times of all profiled metabolites.

### Data processing and statistical analysis

Every experiment was conducted at least in triplicate, and its derived numerical results were reported with means ± standard deviations (SDs) or standard errors of means (SEMs) and the number of data (n) or replications. Statistical comparisons of means with one-way analysis of variant (ANOVA) and other statistical interpretations of regression curves were conducted at 95% confidence level (*α* = 0.05) using Prism version 9.4.0 (GraphPad Software LLC., San Diego, CA, USA), and the significance (*p*) values were reported. For GC-MS data processing, the systematic detection and deconvolution of all peaks derived were caried out using MS-DIAL version 4.7 [52]. The processed peaks were screened and identified as candidate metabolites based on their similarity index cut-off over 70% after an alignment against the National Institute of Standards and Technology (NIST) 14 Mass Spectral Library [53] and the FiehnLib libraries [54]. The candidate metabolites were further analyzed, interpreted, and annotated using MetaboAnalyst version 5.0 (https://www.metaboanalyst.ca). Metabolites showing significant change across different cell types tested were assessed based on their normalized concentrations. The correlation between metabolite profiles of different cell types tested was visualized by sPLS-DA. Specific pathways differently expressed due to their associated metabolites at varying levels were defined using SMPDB (https://www.smpdb.ca) and KEGG (https://www.genome.jp/kegg/) databases and ranked by *p* values and enrichment ratios. The pathway impact was also computed based on the importance measures of individually matched metabolites normalized by those of all metabolites in a mediated pathway, which was presented as a correlation plot with –log *p* values.

## Data Availability

Contact to the corresponding authors for availability.
